# Adaptation mechanism and tolerance of *Rhodopseudomonas palustris* PSB-S under pyrazosulfuron-ethyl stress

**DOI:** 10.1186/s12866-018-1361-y

**Published:** 2018-12-07

**Authors:** Xiang-Wen Luo, De-Yang Zhang, Teng-Hui Zhu, Xu-Guo Zhou, Jing Peng, Song-Bai Zhang, Yong Liu

**Affiliations:** 1 Key laboratory of pest management of horticultural crop of Hunan province, Hunan Plant Protection Institute, Hunan Academy of Agricultural Science, No 726 Second Yuanda Road, Furong District, Changsha, 410125 Hunan province People’s Republic of China; 2 Plant Protection College, Hunan Agricultural University, Changsha, 410128 China; 3 Department of Entomology, University of Kentucky, Lexington, KY 40546 USA

**Keywords:** Pyrazosulfuron-ethyl, *Rhodopseudomonas palustris* PSB-S, Cytology, Proteomic, Adaption mechanism

## Abstract

**Background:**

Pyrazosulfuron-ethyl is a long lasting herbicide in the agro-ecosystem and its residue is toxic to crops and other non-target organisms. A better understanding of molecular basis in pyrazosulfuron-ethyl tolerant organisms will shed light on the adaptive mechanisms to this herbicide.

**Results:**

Pyrazosulfuron-ethyl inhibited biomass production in *Rhodopseudomonas palustris* PSB-S, altered cell morphology, suppressed flagella formation, and reduced pigment biosynthesis through significant suppression of carotenoids biosynthesis. A total of 1127 protein spots were detected in the two-dimensional gel electrophoresis. Among them, 72 spots representing 56 different proteins were found to be differently expressed using MALDI-TOF/TOF-MS, including 26 up- and 30 down-regulated proteins in the pyrazosulfuron-ethyl-treated PSB-S cells. The up-regulated proteins were involved predominantly in oxidative stress or energy generation pathways, while most of the down-regulated proteins were involved in the biomass biosynthesis pathway. The protein expression profiles suggested that the elongation factor G, cell division protein FtsZ, and proteins associated with the ABC transporters were crucial for *R. palustris* PSB-S tolerance against pyrazosulfuron-ethyl.

**Conclusion:**

Up-regulated proteins, including elongation factor G, cell division FtsZ, ATP synthase, and superoxide dismutase, and down-regulated proteins, including ALS III and ABC transporters, as well as some unknown proteins might play roles in *R. palustris* PSB-S adaptation to pyrazosulfuron-ethyl induced stresses. Functional validations of these candidate proteins should help to develope transgenic crops resistant to pyrazosulfuron-ethyl.

**Electronic supplementary material:**

The online version of this article (10.1186/s12866-018-1361-y) contains supplementary material, which is available to authorized users.

## Background

Pyrazosulfuron-ethyl, one of the acetolactate synthase (ALS; EC4.1.3.18) inhibiting herbicides in the sulphonylurea family [1], has been widely used to control weed growth in commercial cereal, soybean, and vegetable fields. Due to its high herbicidal activity (2–100 g/hm^2^), specific plant selectivity, very low aquatic life toxicity, and low bio-concentration in the non-targeted organisms [2, 3], utilization of pyrazosulfuron-ethyl in China has been increased significantly to reduce the labor intensity and increase the input-output ratio [4]. However, pyrazosulfuron-ethyl is also known to be a long lasting herbicide in the agro-ecosystem (*t*_1/2_ > 74.6 d for pyrazosulfuron-ethyl in soil with half maximum water holding capacity) [5], and its residue is toxic to certain food crops and others organisms [6, 7]. This sensitivity limited the potential application of pyrazosulfuron-ethyl in many important food crops.

Chemicals of sulfonylurea family could change the cell structure of mouse pancreatic β-cells and pancreatic islet cells [8, 9]. Sulphonylurea herbicide tribenuron-methyl could change anther cell morphology and resulted in male sterility of rapeseed (*Brassica napus*) and *Arabidopsis* [10]. The plastid ultrastructure was abnormal in pollen mother cells and tapetal cells in male sterility of *Brassica napus* L treated by sulphonylurea herbicide monosulfuron ester sodium [11]. Pyrazosulfuron-ethyl also alter the cell structure of degrading microbacteria [12]. It is rational to deduce pyrazosulfuron-ethyl alter the cell morphology of organism, which should be one of the vital adaptation against pyrazosulfuron-ethyl.

To counteract the toxicity of pyrazosulfuron-ethyl residual in the agro-ecosystem, crops need to be improved to show better tolerance or resistance to pyrazosulfuron-ethyl treatment though various adaptions and/or modifications [13, 14]. To date, only a few genes, including *ALS* genes and *cytochrome P-450* gene, were cloned and characterized to be resistant genes against herbicides in the sulphonylurea family [15–17]. However, successful incorporation of these resistant genes into commercial crops still needs time and effort.

Proteomics is a quick and high throughput technology for identifications of proteins in cells or in tissues grown under various conditions. One of the protomic technologies utilizes two-dimensional gel electrophoresis followed by protein identifications through mass spectrometry. It has been employed by many research groups to uncover the strategies used by plants to combat stresses caused by herbicide applications [18, 19]. To date, this technology has not been used to elucidate the molecular mechanisms controlling the resistance in bacteria to sulphonylurea herbicides, despite of the current knowledge on toxicology of decreasing diversity of soil microbial communities and inhibiting population growth tests to *Azospirillum lipoferum* and *Bacillus megaterium* against sulphonylurea herbicides [20].

Bacterial strains belong to genus *Rhodopseudomonas* are known have excellent capacities of hydrogen production, carbon dioxide fixation and organic compounds degradation [21]. Moreover, *R*. sp. S9–1 was documented with high concentration pyrazosulfuron-ethyl tolerance (upto 800 μg/ml), which probably contributed to its mutant *ALS* gene [22]. However, the adaption mechanism of bacterial strains of *Rhodopseudomonas* under pyrazosulfuron-ethyl stress remained unclear. *R. palustris* PSB-S was isolated and characterized to be resistant to pyrazosulfuron-ethyl at a concentration of 200 μg/mL [23]. In this study, we conducted cytological and protein expression studies using pyrazosulfuron-ethyl treated and non-treated PSB-S cells through electron microscopy and 2-dimensional gel-based comparative proteome. We consider that the results presented in this paper may provide useful information or potential strategies to improve crop sensitivity to this herbicide through molecular manipulations.

## Methods

### Bacterial strain, culture conditions and growth media

*Rhodopseumonas palustris* PSB-S was identified previously (DDBJ/ENA/GenBank accession no. of draft genomic sequence: JHAA00000000) and stored at − 80 °C till use.

Culture medium [24] used in this study contained 2.0 g Sodium L-malatate, 2.0 g Sodium glutamate, 1.0 mg KH_2_PO_4_, 0.5 g NaHCO_3_, 0.2 g MgSO_4_·7H_2_O, 0.1 g CaCl_2_·2H_2_O, 2.0 mg MnSO_4_·H_2_O, 0.5 mg FeSO_4_·7H_2_O, 0.5 mg CoCl_2_·2H_2_O, and 0.5 g yeast extract in one liter deionized H_2_O. For solid medium, 15 g technical grade agar was added to one liter liquid medium. After autoclaving, pyrazosulfuron-ethyl was added to the medium at specific concentrations as stated below.

Approximately 10^9^ cfu/mL cells were inoculated to a 120 ml growth medium in 130 mL serum bottles with airproofed rubber plugs and the cultures were grown in a chamber illuminated at approximately 3000 lx and at 30 ± 1 °C. Growth of the cultures was determined by Spectrophotometry at 660 nm.

### Scanning and transmission electron microscopy (SEM and TEM)

Morphology of *R. palustris* PSB-S cells was determined by SEM. Briefly, freshly prepared and concentrated cell suspensions were fixed and dried before SEM using an JEXL-230 scanning electron microscope (Japan) as described previously [25].

To determine ultrastructural changes in the *R. palustris* PSB-S cell, cells were fixed and then embedded in LR White resin as described [25]. The specimens were sectioned with a Leica EM UC7 Ultramicrotome (Leica Microsystems, Germany). The sections (70 nm thick) were mounted on 600-mesh formvar-coated copper grids, and examined and photographed under a transmission electron microscope (JEM-1230, JEOL, Tokyo, Japan) as described [25].

### Quantification of photopigments in strain PSB-S cells

Photopigments in strain PSB-S cells were extracted using a modified methanol/acetone extraction method [26]. The cells were collected by centrifuging, rinsed and resuspended in ddH_2_O. Sonicated cell broth was extracted with methanol and acetone. The photopigment Carotenoid (Car) was then quantified by the Jassen formula, C = (D·V·f × 10)/2500 [C, Car quantification (mg); V, total volume of extract buffer; f, dilution fold; D, photodensity of Car at the maximum absorption peak]. The photopigment bacteriochlorin (Bchl) was calculated by the Beer-Lambert-Bouguer law, C = D·V·F/(a·L) × 10^3^ [a, extinction coefficient (L/g·cm); L, optical distance (cm)]. Quantification of total photopigments was determined by addition of Car and Bchl.

### Protein extraction

Total protein was extracted from *R. palustris* PSB-S culture cells using a bacterial protein extraction kit (BigBlueInteractive, NY). Concentrations of total protein in extracts were estimated by the Bradford assay [27]. For each treatment, three protein extracts from three different flasks were prepared.

### Protein separation and quantification through 2D-DIGE electrophoresis

The resulting total protein samples were rehydrated in the sample buffer [8 M urea, 2 M thiourea, 0.5% CHAPS, 40 mM Tris-base, 0.02% bromophenol blue, 1.2% DTT, carrier ampholytes 0.52% (*v*/v) Pharmalyte] and separated on non-linear pH 4–7 gradient immobiline DryStrips (17 cm-long) (GE Healthcare Bio-Sciences AB, Beijing). For the second dimension separation, strips were cup-loaded at the anodic side of 12% SDS-PAGE gels (18 × 20 cm) after overnight rehydration at room temperature [28].

### Comparative analysis and protein identification

Gels were stained with Coomassie blue and images of the gels (three gels per sample) were captured using the TyphoonTM 9410 scanner (GE Healthcare) after destaining [54]. Protein spot were quantified based on the digitized staining intensity within the spot boundaries and used for calculations of protein expressions. The normalized expression profile data were then used to statistically determine the expression changes of individual protein spots. Protein spots showing *t* ≤ 0.05 by the Student-T test were considered to be significantly differentially regulated.

The protein identification process was as previously reported [29]. The protein spots of interest were digested in-gel with bovine trypsin, extracted with 0.1% trifluoroacetic acid in 60% acetonitrile, and analyzed by mass spectrometry (4700 Proteomics Analyzer, ABI, CA) equipped with a pulsed N_2_ laser (337 nm). Calibrations were conducted using the standard peptides. All peptide mass fingerprint spectra were internally calibrated with the trypsin autolysis peaks, and all the known contaminants were excluded during the process. The measured tryptic peptide masses were used for a MASCOT (version 2.2) search at the nonredundant NCBI (NCBInr) database and Swissprot database. The peptide mass spectra searching parameters were set as: fragment mass tolerance: ±0.1 Da, fragment mass/mass tolerance: ±0.5 Da, variable modification: oxidation, and fixed modification of cysteine by carboxymethyl (carbamidomethylation, C), and peptide missed cleavage: 1+. Proteins identified by MALDI-TOF/TOF-MS/MS with C.I. % scores above 95% were selected and considered as significant.

### Bioinformatics analysis

The GO enrichment analysis was performed using the Blast2GO [30]. Metabolic pathways of the identified proteins were generated according to the KEGG database (http://www.genome.jp//kegg/). In addition, the deferentially expressed proteins were further analyzed using the Search Tool for the Retrieval of Interacting Genes/Proteins (STRING; http://string.embl.de/) to build a functional protein association network.

### Total RNA preparation and quantitative RT-PCR (qRT-PCR)

Total RNA from cells of *R. palustris* PSB-S was extracted using TRIzol® reagent as instructed (Invitrogen, Beijing). The quality of total RNA samples was assessed by agarose gel electrophoresis and the concentration of total RNA was estimated using a spectrophotometer. cDNA synthesis was performed using an M-MLV RTase cDNA synthesis kit (TranGen, Beijing). Quantitative PCR (qPCR) was performed using the TransStart Green qPCR SuperMix UDG (TranGen, Beijing). The qPCR reaction mixture (20 μL) consisted of 0.5 μL cDNA, 10 μL UDG, F/R primer (0.5 μL/each), and 8.5 μL ddH_2_O. After 2 min incubation at 50 °C, the reaction was set at 95 °C for 10 min followed by 44 cycles of amplification (95 °C for 5 s, 60 °C for 15 s and 72 °C for 10 s). The last step reaction was carried out at 95 °C for 15 s, 65 °C for 5 s and 95 °C for 5 s. Expression level of *ribulose 1,5-bisphosphate carboxylase/oxygenase* (RubisCO) gene [19] was used as the internal control during the study. Relative expression of each gene was determined using the relative quantification (ddCt) method and was based on three biological replicates. All the primers used for qRT-PCR are listed in Additional file 1: Table S2.

### Statistical analysis

All the statistical analyses were performed using the Data Processing System (DPS, version 9.50) [31]. Values are showed as mean ± standard deviation (SD). Samples showing *ρ* < 0.05 were considered to be statistically significant different.

## Results

### Pyrazosulfuron-ethyl inhibited the growth of *R. palustris* PSB-S

**Fig. 1:**
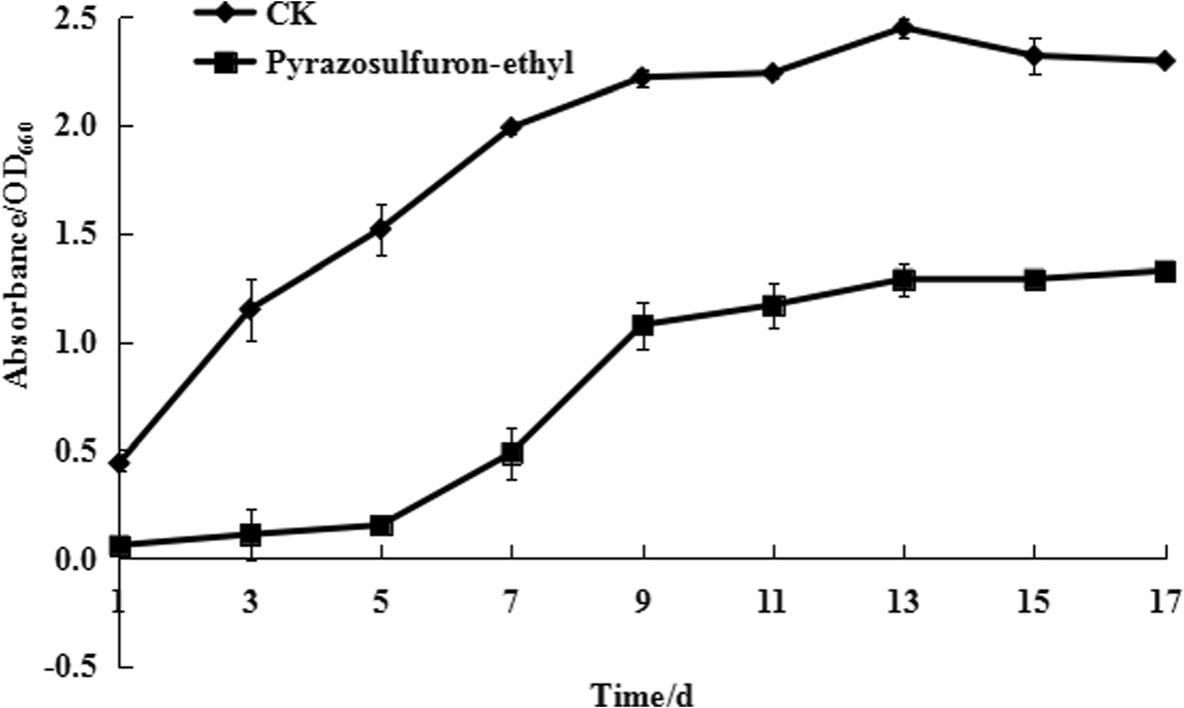
Effect of pyrazosulfuron-ethyl on *R. palustris* PSB-S growth. *R. palustris* PSB-S cultured in the PSB medium without 50 μg/ml pyrazosulfuron-ethyl was used as a control CK. Cell biomass was measured at 1 to 17 days post culturing

The growth of strain PSB-S in PSB medium is shown in Fig. 1. The result of cultivation phase (day 3–11) indicated that the growth of strain PSB-S was significantly inhibited in growth medium containing 50 μg/mL pyrazosulfuron-ethyl, especially during in the exponential growth phase (i.e., day 3–7). The biomass of strain PSB-S cells grown in the PSB medium with 50 μg/mL pyrazosulfuron-ethyl at day 3–7 were only about 15–36% of the cells grown in the PSB medium. During the equilibrium phase of cell growth (i.e., day 7–9), the growth of cells in the PSB medium with 50 μg/mL pyrazosulfuron-ethyl was increased rapidly. After day 9, the biomass of cells grown in the PSB medium with 50 μg/mL pyrazosulfuron-ethyl remained stable till day 11 but still significantly lower than the biomass of cells grown in the PSB medium without pyrazosulfuron-ethyl. Consequently, PSB-S cells were harvested at 7 days post culturing in the PSB medium with or without 50 μg/ml pyrazosulfuron-ethyl and used for further cytological and proteomic analyses.

### Effect of pyrazosulfuron-ethyl on *R. palustris* PSB-S cell morphology

**Fig. 2:**
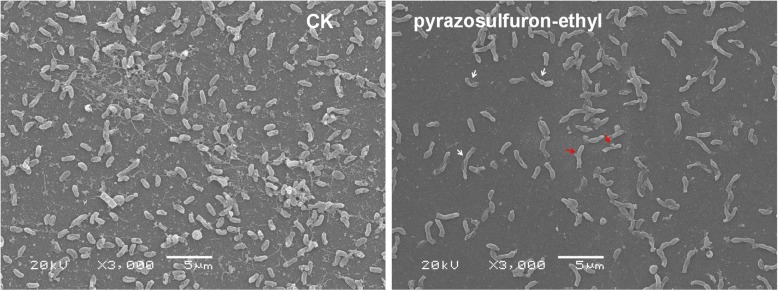
Effect of pyraxosulfuron-ethyl on *R. palustris* PSB-S cell morphology. (a) CK; (b) pyraxosulfuron-ethyl. The cells were harvested at 7 days post culturing and examined by Scanning Electron Microscopy. *R. palustris* PSB-S cells showing curving or budding are indicated with white and red arrows, respectively. Bar = 5 μm

Surface morphology of pyrazosulfuron-ethyl-treated cells was examined by scanning electron microscopy and compared with that shown by the control cells (Fig. 2). Three distinct changes were observed on the surface of pyrazosulfuorn-ethyl-treated bacterial cells. First, pyrazosulfuorn-ethyl treatment inhibited polar flagella generation on bacterial cells. Second, the pyrazosulfuorn-ethyl-treated cells appeared significantly longer (0.74 ± 0.05 μm in diameter and 2.16 ± 0.38 μm in length) than that of the control cells (0.62 ± 0.04 μm in diameter and 3.38 ± 0.54 μm in length). Third, the pyrazosulfuorn-ethyl-treated cells often bent (see white arrows) and budded (see red arrows) while the control cells remained oval or short rod like shapes.

**Fig. 3:**
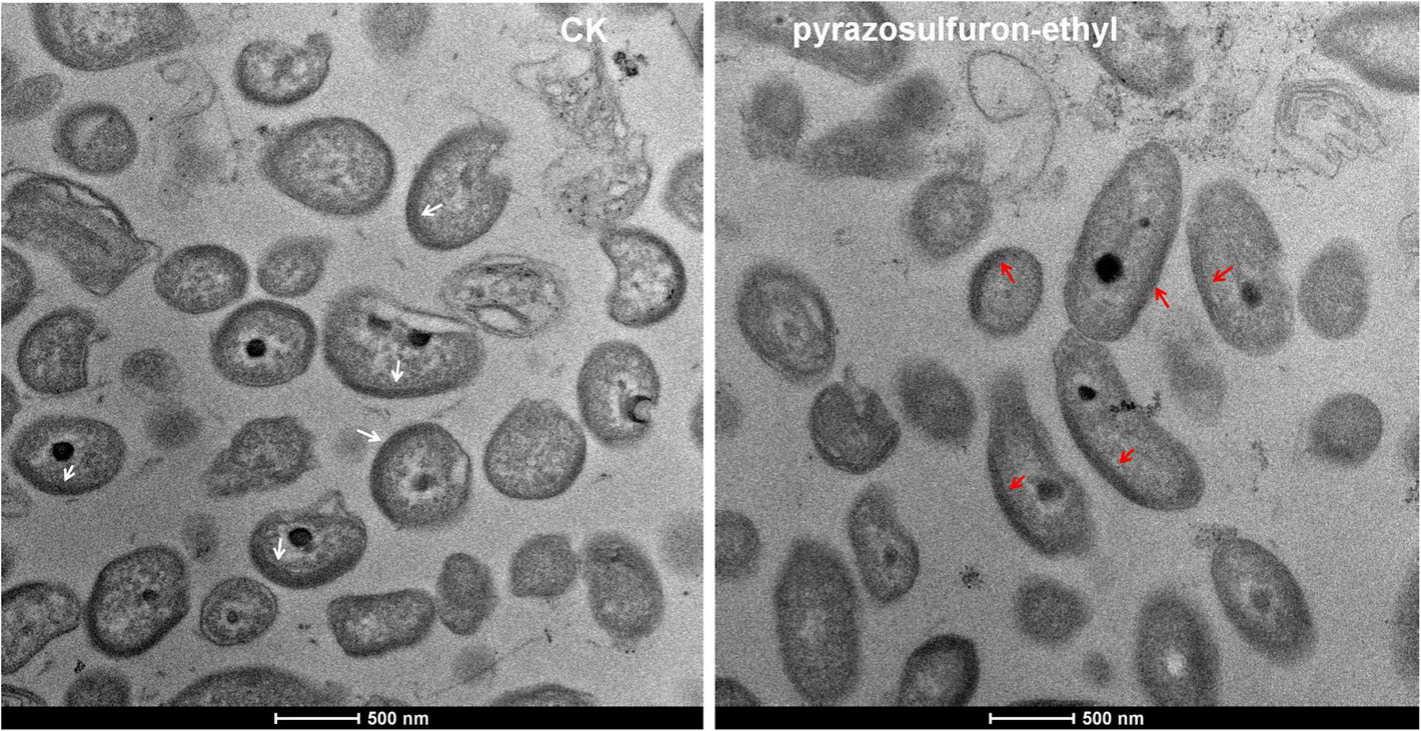
Internal changes in *R. palustris* PSB-S cells treated with pyraxosulfuron-ethyl. (a) CK; (b) pyrazosulfuron-ethyl. *R. palustris* PSB-S cells were fixed and embedded in the resin. Thin sections of the cells were examined for internal changes by TEM. Electron dense areas in the cells are indicated with arrows

Intracellular alterations caused by pyrazosulfuron-ethyl treatment was studied by transmission electron microscopy (TEM). PSB-S cells treated with pyrazosulfuron-ethyl were fixed, embedded and sectioned for TEM. Under the electron microscope, electron dense areas were observed alongside the cell membrane (arrows, Fig. 3). These electron dense areas are known to accumulate lamella photo-pigments. Compared with the control cells, the electron dense areas in the pyrazosulfuron-ethyl-treated cells was smaller, suggesting inhibition of photo-pigments biosynthesis in these cells.

**Fig. 4:**
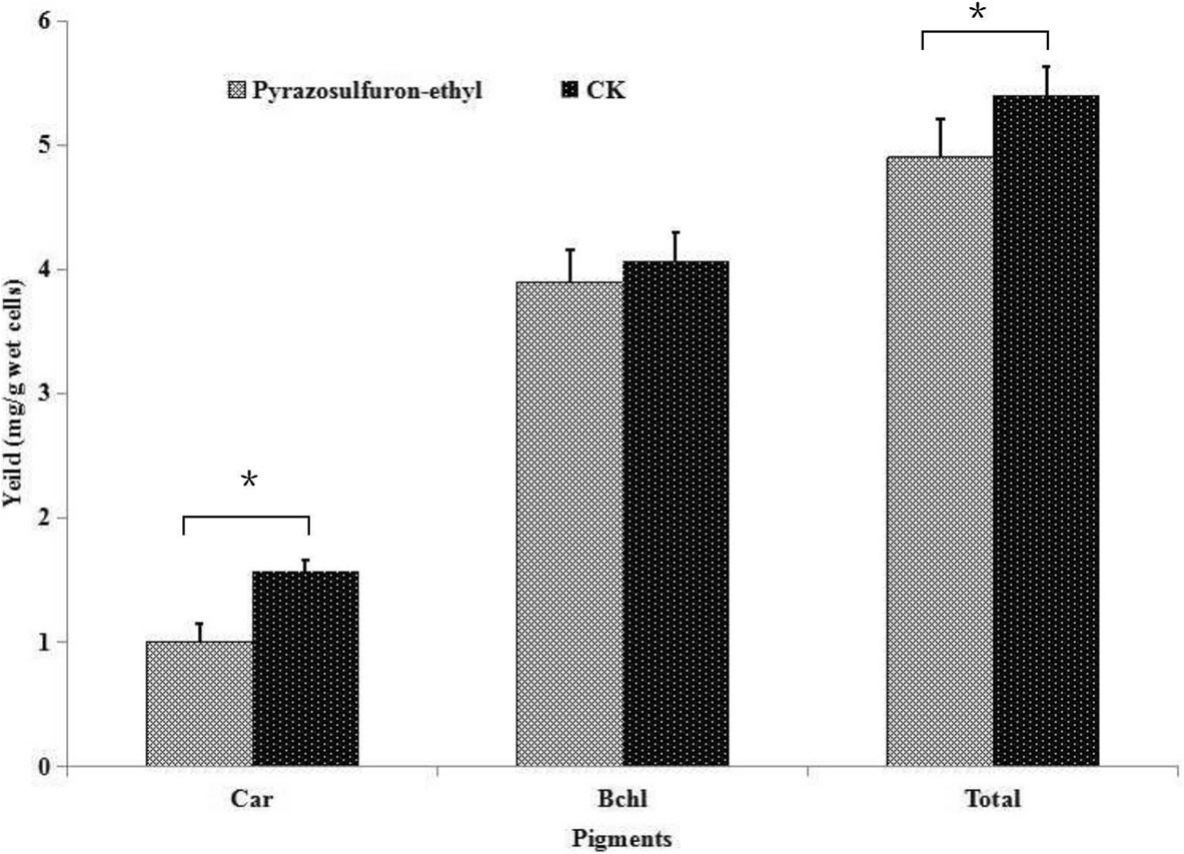
Effect of pyraxosulfuron-ethyl treatment on photo-pigments biosynthesis. Accumulation of carotenoid and bacteriochlorin in the pyraxosulfuron-ethyl treated (pyraxosulfuron-ethyl) or non-treated control (CK) PSB-S cells was measured using a modified methanol/acetone extraction method. Each treatment had three biological replicates. *, *p* < 0.05. Car, Carotenoid accumulation; Bchl, Bacteriochlorin accumulation; Total, total amount of Carotenoid and Bacteriochlorin

Two known photo-pigments, bacteriochlorin and carotenoid, were extracted from the pyrazosulfuron-ethyl-treated or non-treated strain PSB-S cells and quantified (Fig. 4). The result indicated that the accumulation of carotenoid in the pyrazosulfuron-ethyl-treated cells was significantly inhibited by about 23.04% compared with the control cells. The biosynthesis of bacteriochlorin in the pyrazosulfuron-ethyl-treated cells was, however, not affected significantly by the pyrazosulfuron-ethyl treatment. Although the total photo-pigments biosynthesis in the pyrazosulfuron-ethyl-treated cells was inhibited significantly, this inhibition was likely caused by the reduction of carotenoid biosynthesis.

### 2-DE gel and mass spectrometry of protein patterns from *R. palustris* PSB-S cells

**Fig. 5:**
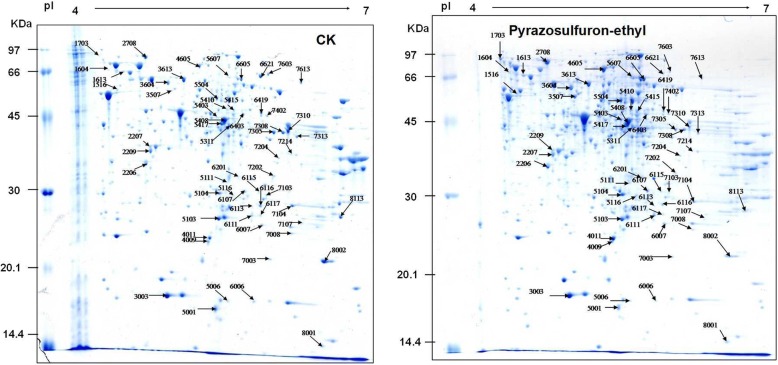
Proteome profiles for pyrazosulfuron-ethyl-treated (pyrazosulfuron-ethyl) or non-treated control (CK) *R. palustris* PSB-S cells. (a) CK; (b) pyrazosulfuron-ethyl. Total protein was isolated from harvested cells and separated through 2-Dimensional Gel Electrophoresis (2DGE). After staining with Coomassie blue, the gels were scanned using the TyphoonTM 9410 scanner. Deferentially expressed protein spots are indicated with arrows and the numbers of the protein spots are shown adjacent to the arrows

To reveal the protein expression changes in *R. palustris* PSB-S cells under pyrazosulfuron-ethyl stress, we extracted total protein from *R. palustris* PSB-S cells treated with 50 μg/mL pyrazosulfuron or non-treated control cells for proteome profile analyses by 2-DE. Protein extracts from three independent biological samples per treatment were visualized individually in three technical replicate gels for comparison. About 1127 detectable protein spots were counted in each gel after Coomassie Brilliant Blue staining (Fig. 5). The three sets of independent biological samples ensured that the changes of protein abundance in cells were reproducible and thus reliable. Analyses of the gel images showed that over 246 protein spots were altered significantly in their expression according to the *t*-test (*t* < 0.05). Of these identified protein spots, 102 spots were suitable for further analyses by Mass Spectrometry. After mass spectrometry, the protein spots were annotated using the Uniprot Knowledgebase (www.uniprot.org) or the NCBI (www.ncbi.nlm.nih.gov) database with BLASTP. Identities of 56 protein spots were successfully identified while the other 46 protein spots remained unidentified mainly due to their lower total ion score [C.I.; < 95% (data not shown)].

Twenty six up- and thirty down-regulated proteins in *R. palustris* PSB-S cells are shown in Additional file 1: Table S1. The protein displaying the highest up-regulation was elongation factor G (gi|169,830,041; + 24.83 fold; protein spot number 1703), followed by a cell division associated protein FtsZ (gi|115,524,129; + 7.49 fold; protein spot 1604) and the ATP synthase subunit alpha (gi|169,826,598; + 3.49 fold; protein spot 3507). The protein showing the strongest down-regulation was a periplasmic component of an ABC-type branched-chain amino acid transport complex (gi|115,525,850; − 0.07 fold; protein spot 7310) followed by a protein with unknown function (hypothetical protein MT1820.1; gi|15,841,238; − 0.13 fold; protein spot 6621).

Ten differential expressed proteins, including five up-regulated and five down-regulated proteins, were selected for validation analyses through quantitative RT-PCR (qRT-PCR) using specific primers (Additional file 1: Table S2). Results of the analyses indicated that the transcriptional levels of the selected genes agreed with the protein expression profiles determined by the proteomic analyses (Additional file 1: Figure S1).

The identified differentially expressed proteins were used to determine the enriched GO categories, including biological processes, molecular functions and cellular localizations. The main enriched categories for the up- and down-regulated proteins are shown in Additional file 1: Fig. S2. The three major groups in the biological processes category contained proteins involved in biological processes, small molecule metabolic processes and biosynthetic processes (Additional file 1: Figure S2A). The four main groups in the cellular localization category were proteins related to cellular component, cell, cytoplasm, and intracellular (Additional file 1: Figure S2B). For the molecular function category, most up-regulated proteins were grouped in the molecular function, ion binding, transferase activity, and oxidoreductase activity groups. The down-regulated proteins were, however, grouped in the molecular function, ion binding, and ATPase activity groups respectively (Additional file 1: Figure S2C).

**Fig. 6:**
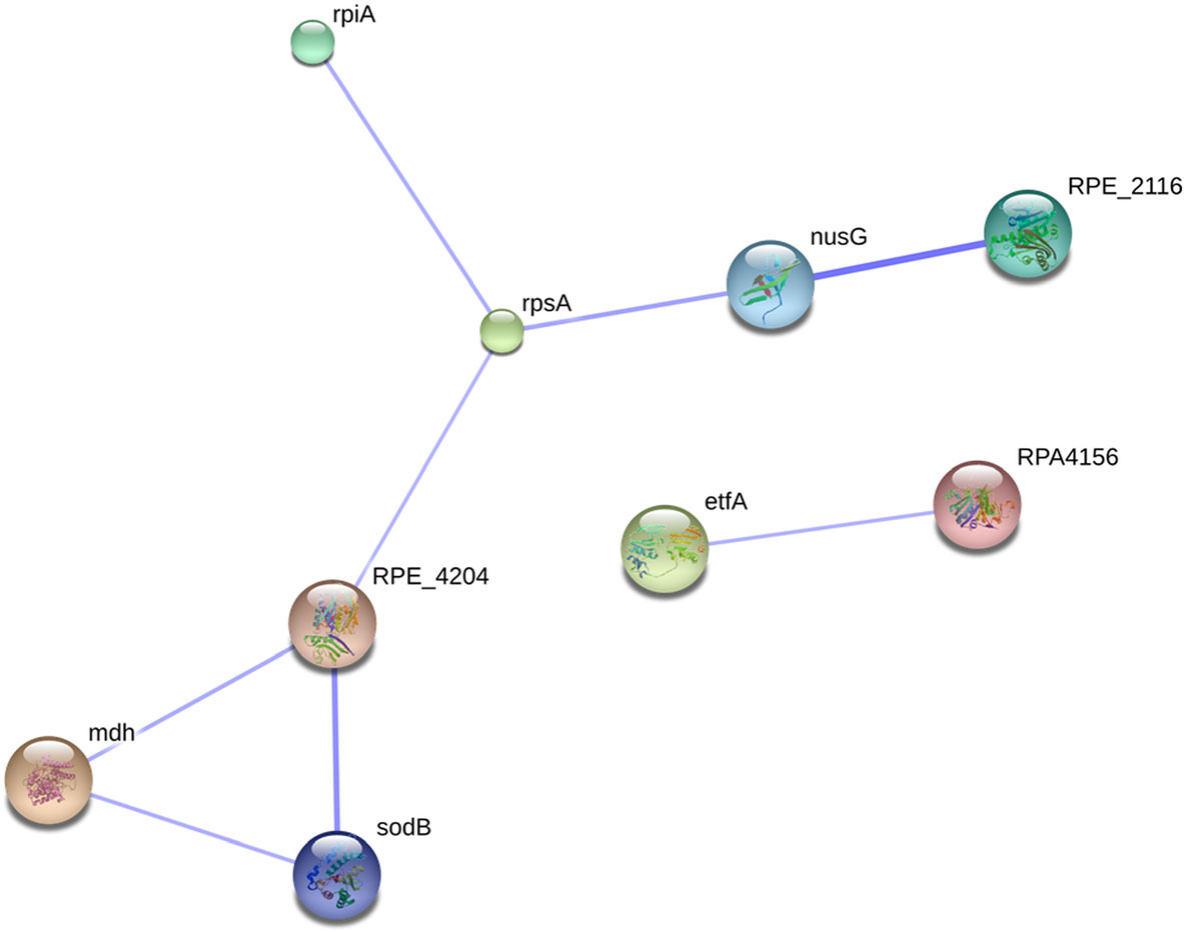
Protein-protein interaction networks predicted for differentially expressed proteins using STRING Database version 10.0

In addition to GO, protein-protein interaction networks were also predicted in this study using STRING Database (http://string-db.org/, version 10.0). As shown in Fig. 6, the deferentially expressed proteins were mainly enriched in the term synthesis and degradation of ketone bodies (RPA4156) and was connected to electron-transfer-flavoprotein (etfA) based on protein homology. Term cysteine and methionine metabolism (RPE_4204) was connected to malate dehydrogenase (mdh) based on protein homology and term cell division (RPE_2116) was linked to gene co-occurance. Term cellular component organization (RPE_2116) was connected to transcription elongation (nusG) as gene co-occurrance.

RPA4156, etfA and their connected proteins are involved in energy generation and homeostasis. These proteins may affect bacterial cell survival. RPE_4204 and mdh, and RPE_2116 and its interacted proteins are known to participate in proteins synthesis and multiplication. These proteins may be crucial for bacterial cells propagation. RPE_2116, nusG and their interacted proteins are known to be responsible for protein translation, biosynthesis and cell structure. These protein may affect bacterium cell morphology.

## Discussion

### Effect of pyrazosulfuron-ethyl on *R. palustris* PSB-S cell cytological changes

Pyrazosulfuron-ethyl was reported to inhibit the activities of cellulolytic, proteolytic and phosphate solubilizing enzymes in soil bacteria [20]. In this study, the cytological changes in *R.* palustris PSB-S cells treated with pyrazosulfuron-ethyl included decrease of biomass and cell size (Fig. 2). These changes may correlate with the 7.49-fold up-regulation of cell division protein FtsZ (protein spot 1604) in the pyrazosulfuron-ethyl-treated cells. It was previously reported that FtsZ protein could regulate the initial peptidoglycan synthesis, inhibit cell division during the onset of cytokinesis, and increase the length of bacterial and archaea cells [32]. Flagella biosynthesis was reported to be controlled by *fla* genes and the cognate CheY protein [33]. In the current study, the expressions of fla proteins and the CheY protein were apparently not affected by pyrazosulfuron-ethyl treatment according to the 2-DE gel analyses (Fig. 5, Additional file 1: Table S1). We speculate that the loss of polar flagella formation on the pyrazosulfuron-ethyl-treated cells was caused by a significant reduction of biomass production in the pyrazosulfuron-ethyl-treated cells. It is also possible that of our 2-DE gel analyses were not sensitive enough to detect the changes of these proteins as previously described [34].

*R. palustris* can proliferate through two major developmental processes (i.e., binary fission under oxygen limitation and illumination conditions or budding) [35]. Because pyrazosulfuron-ethyl treatment could induce *R. palustris* PSB-S cells to bud under the oxygen limitation and illumination conditions, it is possible that the stresses caused by pyrazosulfuron-ethyl treatment perturbed the development of PSB-S cells. The reason why *R. palustris* PSB-S can tolerate pyrazosulfuron-ethyl treatment might be interpreted as the bacteria has evolved both proliferation strategies mentioned above to counteract the toxicity of pyrazosulfuron-ethyl.

### Photopigment biosynthesis and photosynthetic rate

*Rhodopseudomonas* bacteria are purple nonsulfur phototrophic organisms with unique abilities to use light as its energy source for photosynthesis. The photosynthetic reaction complexes of *Rhodopseudomonas* bacteria contain two photopigments (bacteriochlorin b and carotenoid) that can convert carbon dioxide to cell mass [36]. Results obtained in this study showed that pyrazosulfuron-ethyl could significantly inhibit the biosynthesis of carotenoid (Figs. 3 and 4), leading to a decrease in light aggregation capacity [37]. As a compensation, the photosynthetic rate in pyrazosulfuron-ethyl-treated *R. palustris* PSB-S cells was up-regulated (protein spot 3507) (Additional file 1: Table S1, KO00195, http://www.genome.jp/kegg-bin/show_pathway?ko00195). This increased photosynthetic rate may be considered as a strategy used by *R. palustris* PSB-S cells to counteract the reduction of light aggregation. This strategy may serve as a crucial defense mechanism in *R. palustris* PSB-S cells against pyrazosulfuron-ethyl toxicity.

### Cell homeostasis

Maintenance of a relatively constant internal cytosol concentrations under different environmental stresses is essential for most organisms to survive [38]. Pyrazosulfuron-ethyl is known to be hydrophobic [39]. This character may allow it to permeate into cells and change the homeostasis of *R. palustris* PSB-S cells. To counteract the perturbation, *R. palustris* PSB-S cells down-regulated the expressions of proteins belonging to the ABS transporter family (i.e., protein spot 5001, 8113, 6115, 7308, 7313, 7103 and 7310; Additional file 1: Table S1) upon pyrazosulfuron-ethyl treatment. The down-regulation of these ABS transporter family protein expressions might resulted in limitation of pyrazosulfuron-ethyl penetration into cytoplasm through cell membrane [40]. Prevention or limitation of pyrazosulfuron-ethyl penetration into cell may be crucial for *R. palustris* PSB-S to survive under the pyrazosulfuron-ethyl stress.

### Pyrazosulfuron-ethyl inactive target proteins

The active mechanism of herbicides in the sulfonylurea family to kill weeds is to inhibit the catalytic activity of acetolactate synthase (ALS), rather than to inhibit the biosynthesis of ALS [41]. This active mechanism may not apply to the results obtained in this study because our qRT-PCR (Additional file 1: Fig. S1) and proteome (Additional file 1: Table S1) analyses demonstrated that the expression of ALS 3 catalytic subunit (protein spot 7613) and ALS 3 regulatory subunit (protein spot 8002), the large and small subunit of ALS 3 protein complex, were significantly down-regulated.

Plants harboring mutant acetolactate synthase (ALS) genes were shown to be resistant to sulfonylurea herbicides [42–44]. It was also reported that although the activities of *Salmonella typhimurium* ALS II/ALS III or *Escherichai coli* ALS III could be inhibited by sulfometuron-methyl, their ALS I was insensitive to sulfometuron methyl [41, 45]. Like *E. coli*, *R. palustris* ALS I and ALS III are encoded by *ilvB* and *ilvHI*, respectively, while the missed ALS II is encoded by *ilvG* [46, 47]. In this study, the expression of both ALS III subunits were suppressed by pyrazosulfuron-ethyl treatment while the expression of ALS I protein remained unchanged. This finding may explain why *R. palustris* PSB-S is resistant to pyrazosulfuron-ethyl application in field.

In bacteria, the function of ALS is known to involve isoleucine and valine biosynthesis [48]. It is possible that down-regulation of ALS III protein expression in pyrazosulfuron-ethyl-treated cells resulted in an down-regulation of proteins involved in cysteine and methionine metabolism (i.e., RPE_4204). In addition, the expressions of malate dehydrogenase (mdh) and proteins important in cell division (RPE_2116) pathway were also modulated (Fig. 6). ALS was also reported to play a distinct role in sodium-ion homeostasis in plant cells, plant patterning and development [49] as well as isobutanol biosynthesis [50], important for bacteria resistance to environmental stress [51]. Consequently, we speculate that ALS III is a crucial enzyme in metabolic pathway controlling *R. palustris* PSB-S adaption to pyrazosulfuron-ethyl stress.

### Proteins with unknown functions

Five down-regulated proteins were annotated as proteins with unknown functions (protein spot 5006, 6107, 7104 and 7107) or hypothetical protein MT1820.1 (protein spot 6621). Protein spot 5006 sheared partial sequence homology with hypothetical protein blr5132 [52] which was shown to have a conserved domain similar in structure to chorismate mutase important in synthesizing essential amino acids, phenylalanine and tyrosine in bacteria [53, 54]. Protein spot 6107 sheared a conserved domain with enoyl-[acyl-carrier-protein] reductase of *Mycobacterium tuberculosis* [55], a key enzyme in the type II fatty acid synthesis system. Protein spot 7104 sheared sequence homology with 3-oxoacid CoA-transferase subunit A of *Rhodopseudomonas palustris* [36] known to be crucial in energy generation [56]. Protein spot 7107 sheared sequence homology with DNA-binding response regulator. It was reported that suppression of this regulator abolished bacteria growth under phosphate limitation conditions [57]. Down-regulation of these four protein expressions in the pyrazosulfuron-ethyl-treated PSB-S cells might result in inhibition of biosynthesis of essential amino acids and fatty acid, and energy generation leading to a reduction of biomass production in PSB-S cells (Fig. 1). The hypothetical protein MT1820.1 (protein spot 6621) has no known conserved domain. Its cellular localization and biological function also remain obscure. Whether down-regulation of this protein can affect PSB-S cell growth under the pyrazosulfuron-ethyl stress requires further investigation.

## Conclusion

Results presented in this paper showed pyrazosulfuron-ethyl treatment caused significant changes in morphology and photopigment biosynthesis in *R. palustris* PSB-S cells. Changes in proteomic profile in the pyrazosulfuron-ethyl stressed *R. palustris* PSB-S cells are also presented. The up-regulated proteins are mainly involved in transcription, stress response, or small molecule metabolism. Up-regulation of protein expressions, including elongation factor G, cell division FtsZ, and, ATP synthase, and superoxide dismutase, as well as down-regulation of protein expressions, including ALS III and ABC transporters, and other proteins with unkown functions may play roles in *R. palustris* PSB-S survival and adaptation to pyrazosulfuron ethyl stresses. Further functional studies are needed to elucidate the functions of these proteins in bacteria adaption to stresses. The proteins identified through these studies should benefit the generations of transgenic crops resistant to the toxicities of herbicides beloning to the sulphonylurea family.

## Additional file

Additional file 1: **Figure S1.** Comparison of results obtained through protein expression analysis (blue bars) or qRT-PCR (red bars). The height of the bars indicate the fold changes. Identification numbers of the analyzed proteins are indicated. **Figure S2.** Gene ontology (Go) enrichment of the identified up- or down-regulated proteins in *R. palustris* PSB-S cells treated with 50 μg/ml pyrazosulfuron-ethyl. A protein was considered to be differentially expressed in the pyrazosulfuron-ethyl-treated *R. palustris* PSB-S cells if *t* < 0.05. The GO enrichment analyses were performed using Blast2GO. (A) Number of proteins belonging to various groups in the biological process category. (B) Number of proteins belonging to various groups in the molecular function category. (C) Number of proteins belonging to various groups in the cellular localization category. **Table S1.** Differentially expressed proteins during *R.palustris* PSB-S treated with 50 mg/L pyrazosulfuron. **Table S2.** Primers for qRT-PCR. (PDF 293 kb)

## Abbreviations

2-DE: 2-dimensional gel electrophoresis
ALS: acetolactate synthase
Bchl: bacteriochlorin
Car: barotenoid
DPS: data processing system
etfA: electron-transfer-flavoprotein A
GO: gene orthology
KEGG: Kyoto Encyclopedia of Genes and Genomes
MALDI-TOF/TOF-MS: matrix-assisted laser desorption /ionization tandem time-of-flight mass spectrometry
mdh: malate dehydrogenase
RubisCO: ribulose 1,5-bisphosphate carboxylase/oxygenase
SD: standard deviation
TEM: transmission electron microscopy
