# Supplementary material for: Adaptation mechanism and tolerance of Rhodopseudomonas palustris PSB-S under pyrazosulfuron-ethyl stress
Source: BMC Microbiol. 2018 Dec 7;18:207. doi: 10.1186/s12866-018-1361-y (PMC6286529; doi:10.1186/s12866-018-1361-y)
Supplement: Supplementary file 1 — Figure S1. Comparison of results obtained through protein expression analysis (blue bars) or qRT-PCR (red bars). The height of the bars indicate the fold changes. Identification numbers of the analyzed proteins are indicated. Figure S2. Gene ontology (Go) enrichment of the identified up- or down-regulated proteins in R. palustris PSB-S cells treated with 50 μg/ml pyrazosulfuron-ethyl. A protein was considered to be differentially expressed in the pyrazosulfuron-ethyl-treated R. palustris PSB-S cells if t < 0.05. The GO enrichment analyses were performed using Blast2GO. (A) Number of proteins belonging to various groups in the biological process category. (B) Number of proteins belonging to various groups in the molecular function category. (C) Number of proteins belonging to various groups in the cellular localization category. Table S1. Differentially expressed proteins during R.palustris PSB-S treated with 50 mg/L pyrazosulfuron. Table S2. Primers for qRT-PCR. (PDF 293 kb) [file 12866_2018_1361_MOESM1_ESM.pdf]

# **Analysis of adaptation mechanism of *Rhodopseudomonas palustris* PSB-S resistance to pyrazosulfuron-ethyl through cytological and proteomic studies**

**Authors**: Xiangwen Luo[1,2]#, Deyang Zhang[1,2]#, Tenghui Zhu[2], Xuguo Zhou[3], Du Jiao[1], Jing Peng[1,2], Songbai Zhang[1]*, Yong Liu[1,2]*

**Affiliations**:

[1]*Key laboratory of pest management of horticultural crop of Hunan province, Hunan Plant Protection Institute, Hunan Academy of Agricultural Science, Changsha 410125, China*

[2]*Plant Protection College, Hunan Agricultural University, Changsha 410128, China*

[3] *Department of Entomology, University of Kentucky, Lexington, KY, 40546, USA*

***Correspondence** to Yong Liu, haoasliu@163.com; Songbai Zhang, zsongb@hotmail.com

#Contributed equally to this work.

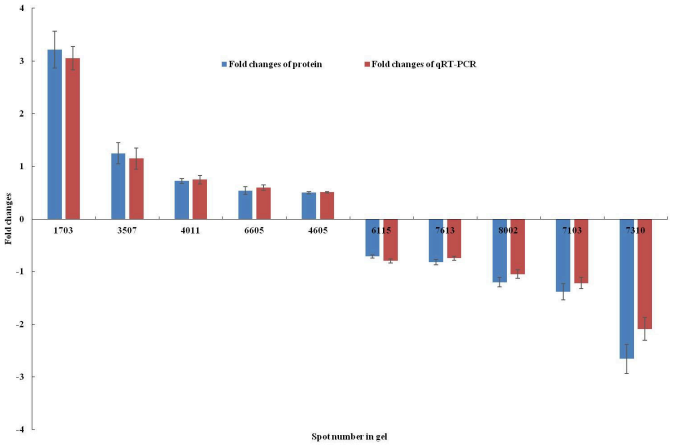


**Figure S1. Comparison of results obtained through protein expression analysis (blue bars) or qRT-PCR (red bars).** The height of the bars indicate the fold changes. Identification numbers of the analyzed proteins are indicated.

A

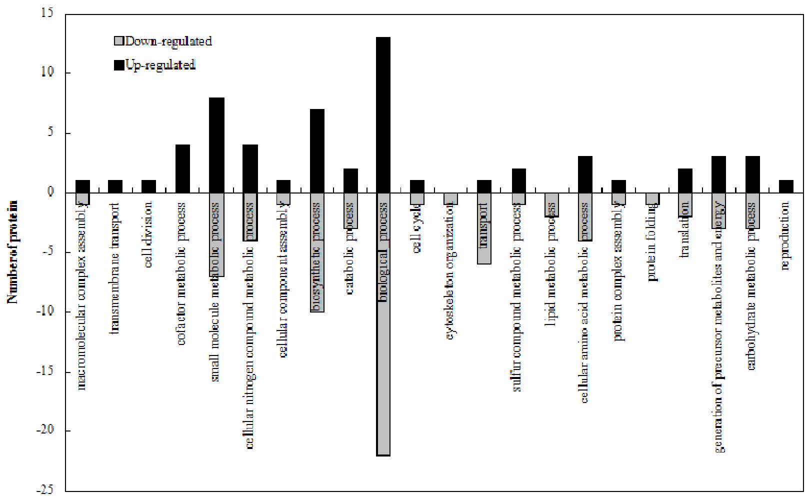

15
10
5
0
-5
-10
-15
-20
-25
Down-regulated
Up-regulated
Number of protein
macromolecular complex assembly
transmembrane transport
cell division
cofactor metabolic process
small molecule metabolic process
cellular nitrogen compound metabolic process
cellular component assembly
biosynthetic process
catabolic process
biological_process
cell cycle
cytoskeleton organization
transport
sulfur compound metabolic process
lipid metabolic process
cellular amino acid metabolic process
protein complex assembly
protein folding
translation
generation of precursor metabolites and energy
carbohydrate metabolic process
reproduction


B

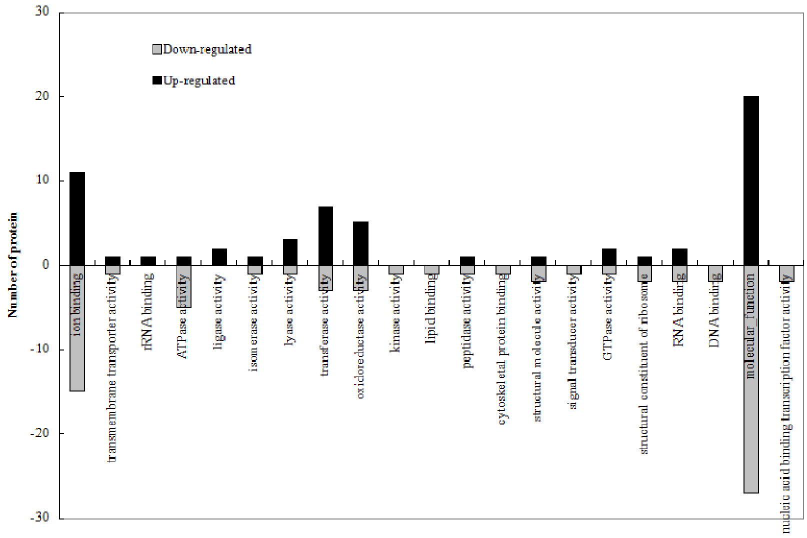

30
20
10
0
-10
-20
-30
Down-regulated
Up-regulated
Number of protein
ion binding
transmembrane transporter activity
rRNA binding
ATPase activity
ligase activity
isomerase activity
lyase activity
transferase activity
oxidoreductase activity
kinase activity
lipid binding
peptidase activity
cytoskeletal protein binding
structural molecule activity
signal transducer activity
GTPase activity
structural constituent of ribosome
RNA binding
DNA binding
molecular_function
nucleic acid binding transcription factor activity


C

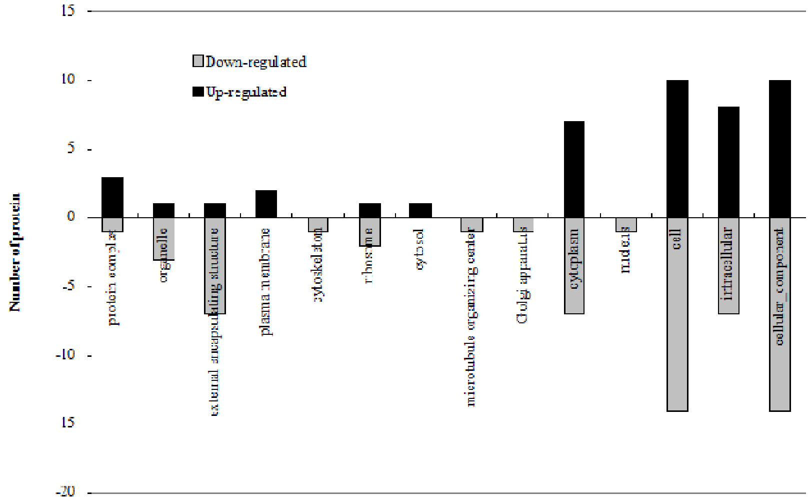

15
10
5
0
-5
-10
15
-20
Down-regulated
Up-regulated
Number of protein
protein complex
organelle
external encapsulating structure
plasma membrane
cytoskeleton
ribosome
cytosol
microtubule organizing center
Golgi apparatus
cytoplasm
nucleus
cell
intracellular
cellular_component

**Figure S2. Gene ontology (Go) enrichment of the identified up- or down-regulated proteins in *R. palustris* PSB-S cells treated with 50 µg/ml pyrazosulfuron-ethyl.** A protein was considered to be differentially expressed in the pyrazosulfuron-ethyl-treated *R. palustris* PSB-S cells if $t < 0.05$. The GO enrichment analyses were performed using Blast2GO. (A) Number of proteins belonging to various groups in the biological process category. (B) Number of proteins belonging to various groups in the molecular function category. (C) Number of proteins belonging to various groups in the cellular localization category.

**Table S1 Differentially expressed proteins during *R.palustris* PSB-S treated with 50 mg/L pyrazosulfuron**

| Spot no/gel[a] | protein name[b] | Acession number[c] | Ion score[d] | Ion CI[e] | Predict Mv(Da)[f] | predict pI[g] | Log(Fold change)[h] |
|---|---|---|---|---|---|---|---|
| Upgrade proteins | | | | | | | |
| 1703 | Elongation factor G | gi\|169830041 | 73 | 99.996 | 76555.51 | 5 | 3.21 |
| 1604 | cell division protein FtsZ | gi\|115524129 | 81 | 99.994 | 62562.12 | 5.13 | 2.01 |
| 3507 | ATP synthase subunit alpha | gi\|169826598 | 185 | 100 | 54683.81 | 5.43 | 1.25 |
| 4011 | superoxide dismutase | gi\|115525836 | 186 | 100 | 22491.03 | 5.9 | 0.72 |
| 2206 | electron transfer flavoprotein subunit alpha | gi\|115526836 | 97 | 100 | 32423.13 | 5.15 | 0.64 |
| 5116 | Ribose-5-phosphate isomerase A | sp\|Q07Q53\|RPIA_RHOP5 | 76 | 99.999 | 25256.45 | 5.51 | 0.63 |
| 5415 | O-acetylhomoserine/O-acetylserine sulfhydrylase | gi\|91975819 | 47 | 92.203 | 46698.58 | 6.24 | 0.61 |
| 5607 | Succinate dehydrogenase flavoprotein subunit | sp\|P31038\|DHSA_RICPR | 54 | 99.727 | 65916.47 | 7.36 | 0.60 |
| 5403 | S-adenosylmethionine synthase | sp\|Q89HP5\|METK_BRAJA | 44 | 97.496 | 43613.24 | 5.88 | 0.57 |
| 6605 | isocitrate lyase | gi\|146340697 | 71 | 99.92 | 61268.64 | 6.01 | 0.54 |
| 2207 | lysine-arginine-ornithine-binding periplasmic protein | gi\|90424048 | 50 | 93.86 | 38065.74 | 8.74 | 0.53 |
| 4605 | Acetyl-coenzyme A synthetase | sp\|Q07VK4\|ACSA_RHOP5 | 43 | 97.254 | 72559.08 | 5.58 | 0.50 |
| 6201 | short-chain dehydrogenase/reductase SDR | gi\|86750316 | 160 | 100 | 32186.35 | 6.01 | 0.47 |
| 5504 | homospermidine synthase | gi\|91975330 | 72 | 99.947 | 53077.3 | 5.67 | 0.46 |
| 2708 | Elongation factor G | sp\|B3QBY3\|EFG_RHOPT | 85 | 100 | 75837.92 | 5.27 | 0.44 |
| 3613 | chaperonin GroEL | gi\|91978588 | 168 | 100 | 57817.73 | 5.46 | 0.44 |
| 2209 | Malate dehydrogenase | sp\|Q07UX5\|MDH_RHOP5 | 67 | 99.992 | 34178.8 | 5.55 | 0.44 |
| 5313 | fructose-1,6-bisphosphate aldolase | gi\|148257629 | 59 | 99.141 | 39478.7 | 5.81 | 0.44 |

| | | | | | | | |
|---|---|---|---|---|---|---|---|
| 1516 | Aspartyl/glutamyl-tRNA(Asn/Gln) amidotransferase subunit B | sp\|A9ISC3\|GATB_BART1 | 40 | 93.25 | 55467.1 | 5 | 0.43 |
| 5311 | phosphoglycerate kinase | gi\|90426241 | 70 | 99.945 | 41864.11 | 5.95 | 0.43 |
| 5408 | S-adenosylmethionine synthetase | gi\|115526198 | 122 | 100 | 43207.87 | 5.88 | 0.43 |
| 3003 | 30S ribosomal protein S6 | sp\|Q3SRY9\|RS6_NITWN | 59 | 99.895 | 15945.16 | 6.45 | 0.43 |
| 1613 | 30S ribosomal protein S1 | sp\|Q6NDP1\|RS1_RHOPA | 76 | 99.998 | 62818.75 | 5.29 | 0.43 |
| 5111 | StAR-related lipid transfer protein 9 | sp\|Q9P2P6\|STAR9_HUMAN | 39 | 92.166 | 521839.66 | 5.91 | 0.42 |
| 6306 | fructose-1,6-bisphosphate aldolase | gi\|86748075 | 98 | 100 | 39550.75 | 5.97 | 0.41 |
| 5417 | putative leucine/isoleucine/valine-binding protein | gi\|91978027 | 157 | 100 | 44363.73 | 6.38 | 0.41 |
| Downgrade proteins | | | | | | | |
| 5006 | unnamed protein product | gi\|27380243 | 147 | 100 | 20377.56 | 8.86 | -0.71 |
| 5001 | ferric uptake regulator family protein | gi\|148251668 | 80 | 99.993 | 17432.8 | 5.7 | -0.71 |
| 6107 | unnamed protein product | gi\|39934274 | 86 | 99.998 | 29441 | 5.9 | -0.76 |
| 3604 | D-3-phosphoglycerate dehydrogenase | gi\|90425581 | 72 | 99.96 | 56105.87 | 5.87 | -0.78 |
| 5103 | ATP-dependent Clp protease proteolytic subunit | sp\|A9ISA4\|CLPP_BART1 | 51 | 99.278 | 23574.88 | 5.72 | -0.80 |
| 8001 | histidine triad (HIT) protein | gi\|91977514 | 127 | 100 | 15759.1 | 6.39 | -0.80 |
| 7003 | transcription antitermination protein NusG | gi\|115525607 | 184 | 100 | 19932.37 | 6.36 | -0.82 |
| 7402 | beta alanine--pyruvate transaminase | gi\|115522940 | 62 | 99.648 | 48049.44 | 6.28 | -0.82 |
| 6111 | isopropylmalate isomerase small subunit | gi\|91974978 | 106 | 100 | 22495.41 | 5.29 | -0.84 |
| 6007 | phosphoglyceromutase | gi\|115522370 | 133 | 100 | 22939.09 | 6.32 | -0.87 |
| 6419 | glutamate dehydrogenase, partial | gi\|29122978 | 127 | 100 | 38019.55 | 7.03 | -0.87 |
| 8113 | ABC-type branched-chain amino acid | gi\|83311427 | 62 | 99.587 | 25796.56 | 6.45 | -0.89 |

| | | | | | | | |
|---|---|---|---|---|---|---|---|
| | transport systems, ATPase component | | | | | | |
| 6115 | ABC transporter-like protein | gi\|91977599 | 78 | 99.991 | 27479.41 | 5.75 | -0.89 |
| 7214 | 30S ribosomal protein S2 | sp\|Q215E9\|RS2_RHOPB | 43.59 | 95.993 | 36323.83 | 6.47 | -0.94 |
| 7305 | branched-chain amino acid ABC transporter | gi\|148256992 | 111 | 100 | 45483.64 | 6.61 | -0.94 |
| 4009 | ubiquinol-cytochrome C chaperone | gi\|90424171 | 66 | 99.807 | 20259.22 | 6.59 | -0.99 |
| 7104 | unnamed protein product | gi\|39937216 | 156 | 100 | 25808.4 | 7.03 | -1.02 |
| 7308 | ABC transporter periplasmic branched chain amino acid binding protein | gi\|115526215 | 152 | 100 | 45133.15 | 7.66 | -1.02 |
| 7613 | acetolactate synthase 3 catalytic subunit | gi\|148257335 | 80 | 99.994 | 64316.3 | 6.2 | -1.05 |
| 7202 | ATPase | gi\|146342585 | 112 | 100 | 32307.3 | 6.14 | -1.11 |
| 7204 | thiosulfate-binding protein | gi\|90425493 | 169 | 100 | 36850.11 | 7.75 | -1.11 |
| 7313 | ABC transporter substrate binding protein | gi\|148258710 | 69 | 99.935 | 41990.6 | 6.62 | -1.11 |
| 7008 | adenine phosphoribosyltransferase | gi\|90425859 | 166 | 100 | 21406.32 | 6.9 | -1.11 |
| 7107 | unnamed protein product | gi\|39934752 | 162 | 100 | 22635.76 | 5.99 | -1.14 |
| 7603 | 1-deoxy-D-xylulose-5-phosphate synthase | sp\|B3QFY7\|DXS_RHOPT | 128 | 100 | 68874.5 | 6.27 | -1.14 |
| 8002 | acetolactate synthase 3 regulatory subunit | gi\|148257333 | 68 | 99.928 | 19991.49 | 6.43 | -1.20 |
| 7103 | ABC transporter | gi\|86749880 | 111 | 100 | 27297.38 | 6 | -1.39 |
| 7304 | extracellular ligand-binding receptor | gi\|86751788 | 183 | 100 | 42536.99 | 7.67 | -1.43 |
| 6621 | hypothetical protein MT1820.1 | gi\|15841238 | 52 | 96.924 | 46162.97 | 6.04 | -2.04 |
| 7310 | ABC-type branched-chain amino acid transport systems periplasmic component | gi\|115525850 | 104 | 100 | 48501.43 | 8.62 | -2.66 |

[a]Number refer to protein designations in Figure5.

[b]Names of identified proteins according to the NCBI (http://www.ncbi.nlm.nih.gov/) or the Uniprot Knowledgebase (http://www.uniprot.org)

[c]Acession number according to the NCBI (http://www.ncbi.nlm.nih.gov/) or the Uniprot Knowledgebase (http://www.uniprot.org)

[d]Ion score

[e]Ion C.I.%

[f]Theoretical molecular mass (Da), calculated from the corresponding amino acid sequence

[g]Theoretical pI, predicted from the corresponding amino acid sequence according to the NCBI (http://www.ncbi.nlm.nih.gov/) or the Uniprot Knowledgebase (http://www.uniprot.org)

[h]Proteins defferetially expressed by *R.palustris* PSB-S cultivated with 50 mg/L pyrazosulfuron compared with without pyrazosulfuron

**Table S2 Primers for qRT-PCR**

| Spot in gel | Protein name | Prime name | Primer sequence (5'--3') |
|---|---|---|---|
| 1703 | Elongation factor G | S1F | ATCGGTTCGGAGAACAACTTCA |
| | | S1R | CGGATCAGACGCTTGAGGG |
| 3507 | ATP synthase subunit alpha | S2F | AGCCAGAAGCTCTACTGCGTTTA |
| | | S2R | CTGCTTGGACAAGTCGTCATAGA |
| 4011 | superoxide dismutase | S3F | GGCTTCGAGAAGTTCAAGACCG |
| | | S3R | TCGACGAACGCCTTCAGGTAG |
| 6605 | isocitrate lyase | S4F | CAAGGACGTCAGCAAGTACAACC |
| | | S4R | CGCTGCACGTTCTTAACATAACC |
| 4605 | Acetyl-coenzyme A synthase | S5F | GACCGAGTGGCCATCTACAT |
| | | S5R | CAGCCAGCTCCTTCAGGTTCACAA |
| 6115 | ABC transporter-like protein | S6F | AGCCGTTCTTTTCCTGCCGTGACC |
| | | S6R | GCCTGCGCCGGCGAAATAGTT |
| 7613 | acetolactate synthase 3 catalytic subunit | S7F | CACCGCCTTACCCCTGTTAG |
| | | S7R | ATGCCGTTCGTCAAATA |
| 8002 | acetolactate synthase 3 regulatory subunit | S8F | AAGGGCCGAGAAAAGTGGTT |
| | | S8R | TAGTATAGATCGGGGTGCTG |
| 7103 | ABC transporter | S9F | CGCCGCGCCGTTCTT |
| | | S9R | GCAGCGTCGAGGTCTTG |
| 7310 | ABC-type branched-chain amino acid | S10F | TCACCGGCGACACCCAGA |
| | | S10R | GCCCGCAACGCCCGACG |
| Reference gene | RubisCO | S11R | ATCCGCAACACGCTGAACGCCGACAA |
| | | S11R | CGACCAGGGTCTTGATCACCGCCAGG |
